# Coat color inheritance in American mink

**DOI:** 10.1186/s12864-023-09348-8

**Published:** 2023-05-04

**Authors:** Persia Carol Thapa, Duy Ngoc Do, Ghader Manafiazar, Younes Miar

**Affiliations:** Department of Animal Science and Aquaculture, Dalhousie University, Truro, NS B2N 5E3 Canada

**Keywords:** American mink, Color inheritance, Mendelian ratio

## Abstract

**Background:**

Understanding the genetic mechanisms underlying coat color inheritance has always been intriguing irrespective of the animal species including American mink (*Neogale vison*). The study of color inheritance in American mink is imperative since fur color is a deterministic factor for the success of mink industry. However, there have been no studies during the past few decades using in-depth pedigree for analyzing the inheritance pattern of colors in American mink.

**Methods:**

In this study, we analyzed the pedigree of 23,282 mink extending up to 16 generations. All animals that were raised at the Canadian Center for Fur Animal Research (CCFAR) from 2003 to 2021 were used in this study. We utilized the Mendelian ratio and Chi-square test to investigate the inheritance of Dark (9,100), Pastel (5,161), Demi (4,312), and Mahogany (3,358) colors in American mink.

**Results:**

The Mendelian inheritance ratios of 1:1 and 3:1 indicated heterozygous allelic pairs responsible for all studied colors. Mating sire and dam of the same color resulted in the production of offspring with the same color most of the time.

**Conclusion:**

Overall, the results suggested that color inheritance was complex and subjected to a high degree of diversity in American mink as the genes responsible for all four colors were found to be heterozygous.

**Supplementary Information:**

The online version contains supplementary material available at 10.1186/s12864-023-09348-8.

## Introduction

American mink (*Neogale vison*) is a semi-aquatic and carnivorous species belonging to the Mustelidae family, and a native species to North America [1]. The fur produced by American mink is one of the most desirable furs due to the astonishing variation in color and high quality, which resulted in its domestication in the late 1800s in Canada [2]. The domestication and breeding of mink in captivity is primarily focused on the production of excellent-quality fur [3, 4]. Due to the increase in demand for fur, farmed mink have been bred intensively for selected traits such as fur color, size, and temperament [5, 6]. This increase in demand in the past decades can be attributed to the color, shades, and texture of their fur [7]. Thus, to meet the demand for fur, mink have been bred to produce an extensive range of colors.

Other than color variation, characteristics of pelt size, color purity, and fur quality also play significant roles in determining the price of a pelt [8–10]. It is clear and understandable that production is influenced by the market demand for a specific color or color combination. Consequently, some mink farmers may focus on producing a particular color of fur, whereas others may cross different color types [11]. Selective breeding of farmed mink has resulted in a broad range of colors commonly introduced as color phases [12].

The color phases are believed to have arisen due to genetic mutations and farming practices [13, 14]. Presently, more than 35 color variations and their combinations have resulted in more than 100 color types in American mink [14]. The immense interest in understanding the mechanism of color mutation led to several genomic studies [13–18]. Until now, out of the total color variations seen in American mink, only eight have been linked with specific DNA mutations [18]. During the last decade, most studies have focused on identifying specific mutant genes rather than investigating the color inheritance using pedigree-based approach in American mink.

However, studying the genetic basis of color inheritance is challenging as an in-depth pedigree expanding up to several generations with the exact color identification and recording is required. One prominent study by Shackelford [19] investigated six coat colors (Royal Silver, Colmira, Ebony, Aleutian, Green Eyed Pastel, and Goofus) in ranch-based American mink and differentiated dominant with recessive color types. Over the past decades, no research has been conducted using pedigree to understand the influence of crossbreeding on the inheritance of specific colors. The genotypes of nine colors of American mink including Pastel, raised in Poland were identified based on the similarity of colors in other mammalian species [20], yet the color inheritance in mink remains unclear.

**Fig. 1:**
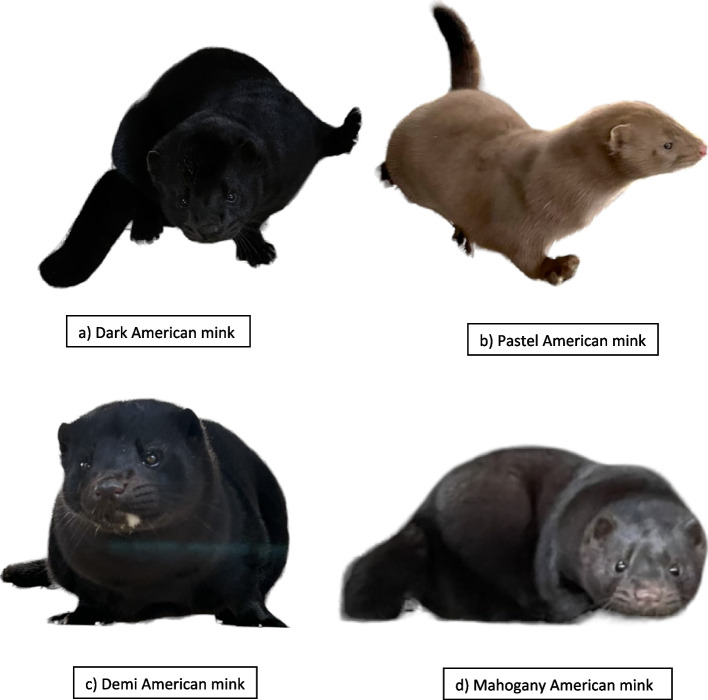
Photographs of American mink showing four types of coat colors

This study aimed to investigate the inheritance of four colors (Dark, Pastel, Demi, and Mahogany) in ranch-based American mink (Fig. 1). The objectives of this pedigree-based study were to 1) investigate the crosses responsible for producing a specific color type under different scenarios and, 2) determine the allelic pair (homozygous or heterozygous) responsible for color type.

## Materials and methods

### Animal care and managements

The research work was approved by the Dalhousie University Animal Care and Use Committee (certification# 2018–009 and 2019–012). The mink used in this study were raised according to the Code of Practice for the Care and Handling of Farmed Mink guidelines published by the Canada Mink Breeders Association [21] at the Canadian Center for Fur Animal Research (CCFAR), Dalhousie University, Faculty of Agriculture (Truro, Nova Scotia, Canada). Mink were housed individually under standard farming conditions, and their diets were regulated according to the production cycle. Each annual production cycle (early March) started with mating between selected males and females. These males and females were selected (late November) based on criteria such as fur grade, disease history, weight, and litter size determining if animals were either used for pelting or breeding. The breeding males and females were selected from the same population and no new mink were introduced in the farm for the sole purpose of breeding. Ideally, two mink (a male & a female) were kept in a single cage with ad libitum feed. However, in the CCFAR, this was not practiced all the time. Some of them were housed separately for feeding measurements. After the birth of the kits (approximately 6–8 weeks), the kits were separated from the dam and housed either in pairs or multiples (mostly same litter).

### Color recording

Since mink fur quality is one of the main selection criteria, grading of fur quality in CCFAR was usually performed on live mink in November or early December each year. Grading requires a physical examination of fur where the guidelines provided by the North American Fur Auctions (NAFA) were followed by an experienced technician [22]. The characteristics such as texture, density, nap length, and color were assessed during grading. The color of a mink was recorded two times during its lifetime: at weaning (6–8-week-old) and based on live grading in November. The color recorded during grading was assigned as the final color type.

### Data collection

A pedigree containing 23,282 mink raised from 2003 to 2021 was used in this study. There were eleven different color types in the pedigree, however, only four of them were used in this study. The four colors were selected since a) they had the highest frequency (more than 3,000 animals per coat color) compared to other colors which were less than 500, and b) these four colors had complete information available regarding the color of ancestors until four generations (Additional File 1).

Accordingly, 21,931 out of 23,282 mink were included in the study. There were 9,100 Dark, 5,161 Pastels, 4,312 Demi, and 3,358 Mahogany in the pedigree. These 21,931 minks were from 1,403 sires and 3,533 dams in the pedigree data, tracing back to 16 generations.

### Statistical methods

The pedigree data was inspected for all four colors extending up to four generations to investigate ancestral color. The animals were grouped into three categories to evaluate different types of mating strategies practiced at the farm. These categories were a) when the coat color of offspring is similar to the color of the sire, b) when the coat color of offspring is similar to the color of the dam, and c) when both of the parents (sire and dam) and offspring have the same color. While grouping animals based on three categories, those animals were not included if the color of either parent was unknown.

For the statistical analysis, the chi-squared test was used in R software version 4.2.0 using the function chisq—test [23]. Assessment of ancestral background until four generations revealed that Dark and Pastel were the only color types with pure backgrounds (all ancestors of the same color). Thus, it was hypothesized that those color types were homozygous and produced only one colored phenotype when crossed based on Mendelian inheritance. On the other hand, the heterozygous pair of gene was tested using the following two Mendelian principles.

The two principles of Mendelian inheritance were considered to determine the allelic pairs responsible for colors. Firstly, genes in charge of the specific color were considered heterozygous (either sire or dam) and the other parent was considered recessive homozygous (i.e., Aa x aa). Secondly, when both parents were considered heterozygous (Aa × Aa). The expected 1:1 (from crossing Aa x aa) and 3:1 (from crossing Aa × Aa) among the offspring based on the Mendelian ratio show a cross between heterozygote with homozygote parent and crosses between two heterozygous parents, respectively [24, 25].

## Results

**Fig. 2:**
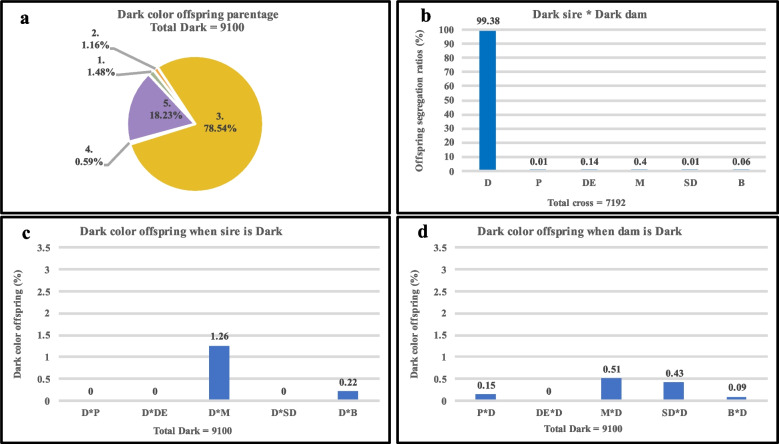
Types of crosses involved in the production of Dark-colored American mink. **a**. The overall parentage of Dark-colored offspring where; 1 = Sire is Dark, 2 = Dam is Dark, 3 = Both sire and dam are Dark, 4 = Neither sire nor dam is Dark, and 5 = Color not identified in sire or dam or both. **b.** mating sire and dam of the Dark color in American mink. **c.** production of Dark color offspring when sire is Dark, and dam is of a different color. **d.** production of Dark color offspring when dam is Dark, and sire is of a different color. D = Dark, P = Pastel, DE = Demi, M = Mahogany, SD = Stardust, and B = Brown

**Fig. 3:**
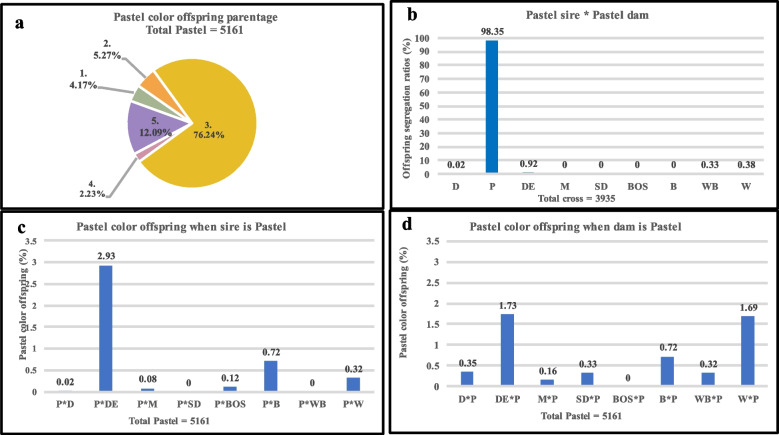
Types of crosses involved in the production of Pastel-colored American mink. **a**. The overall parentage of Pastel-colored offspring where; 1 = Sire is Pastel, 2 = Dam is Pastel, 3 = Both sire and dam are Pastel, 4 = Neither sire nor dam is Pastel, and 5 = Color not identified in sire or dam or both. **b.** mating sire and dam of the Pastel color in American mink. **c.** production of Pastel color offspring when sire is Pastel, and dam is of a different color. **d.** production of Pastel color offspring when dam is Pastel, and sire is of a different color. D = Dark, P = Pastel, DE = Demi, M = Mahogany, SD = Stardust, BOS = Breath of Spring, B = Brown, WB = Winter Blue, and W = White

**Fig. 4:**
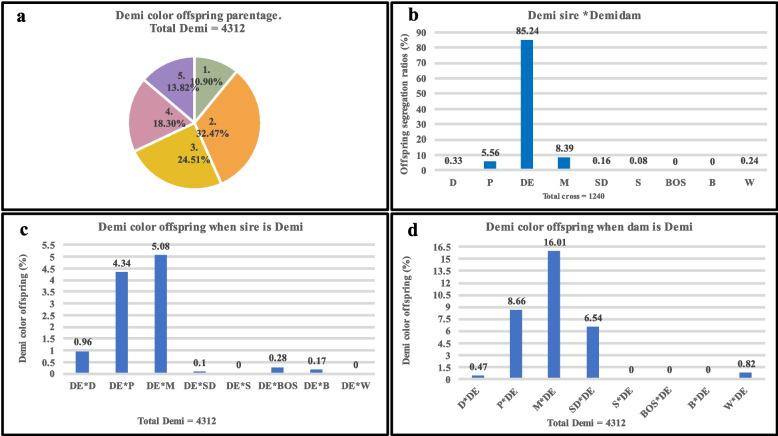
Types of crosses involved in the production of Demi-colored American mink. **a**. The overall parentage of Demi-colored offspring where; 1 = Sire is Demi, 2 = Dam is Demi, 3 = Both sire and dam are Demi, 4 = Neither sire nor dam is Demi, and 5 = Color not identified in sire or dam or both. **b.** mating sire and dam of the Demi color in American mink. **c.** production of Demi color offspring when sire is Demi and dam is of a different color. **d.** production of Demi color offspring when dam is Demi and sire is of a different color. D = Dark, P = Pastel, DE = Demi, M = Mahogany, SD = Stardust, BOS = Breath of Spring, B = Brown, W = White, and S = Sapphire

**Fig. 5:**
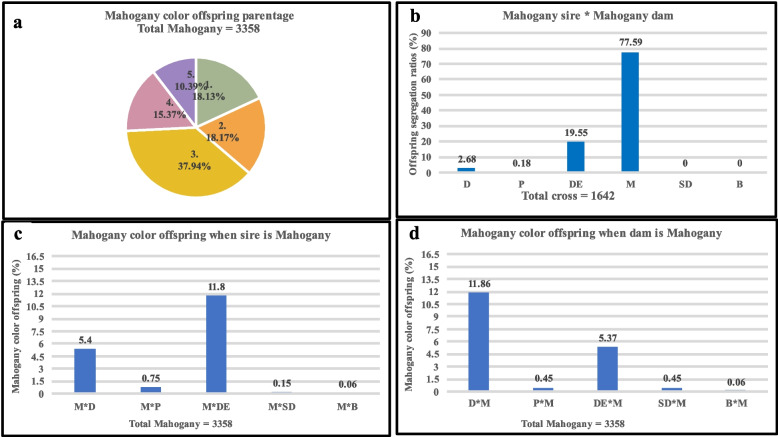
Types of crosses involved in the production of Mahogany-colored American mink. **a**. The overall parentage of Mahogany-colored offspring where; 1 = Sire is Mahogany, 2 = Dam is Mahogany, 3 = Both sire and dam are Mahogany, 4 = Neither sire nor dam is Mahogany, and 5 = Color not identified in sire or dam or both. **b.** mating sire and dam of the Mahogany color in American mink. **c.** production of Mahogany color offspring when sire is Mahogany and dam is of a different color. **d.** production of Mahogany color offspring when dam is Mahogany and sire is of a different color. D = Dark, P = Pastel, DE = Demi, M = Mahogany, SD = Stardust, B = Brown

The overall parentage of Dark, Pastel, Demi, and Mahogany colored individuals in the pedigree is shown in Figs. 2, 3, 4, and 5, respectively. The pie charts (Figs. 2a, 3a, 4a, and 5a) depict the overall parentage of all four-color types under five different conditions. These conditions are: 1 = Sire is of the same color as offspring, 2 = Dam is of the same color as offspring, 3 = Both sire and dam of the same color as offspring, 4 = Neither sire nor dam is of the same color as offspring, and 5 = Color not identified in sire or dam or both. Dark, Pastel, and Mahogany colors were produced more frequently when both of the parents were of the same color. On the contrary, Demi colored offspring were produced more frequently (32.47%) when Demi color dams were used (Fig. 4a). The least number of offspring (0.59% Dark and 2.23% Pastel) were produced when none of the parents were of the same color. In the case of Demi coloration, the overall parentage suggests that few Demi-colored offspring (10.90%) were produced when used as sires. When both parents of Mahogany color were mated, highest numbers of Mahogany colored offspring (37.947%) were produced.

The mating of two parents of Dark, Pastel, Demi, and Mahogany color is shown in Figs. 2b., 3b., 4b., and 5b. respectively. The offspring of two Dark parents resulted in the production of 99.38% Dark, and 0.62% of other colors (Fig. 2b). The parentage of Pastel color individuals also followed a similar pattern as Dark color individuals, where 98.35% of Pastel and 1.65% of other colors were observed (Fig. 3b). Mating two Demi parents resulted in the segregation of offspring, where 85.24% of the offspring were Demi, 8.39% were Mahogany, and 6.37% were others (Fig. 4b). Mating two Mahogany parents resulted in the segregation of offspring where, 77.59% Mahogany, followed by 19.55% Demi and 2.86% other color were observed (Fig. 5b).

The production of variety of colored offspring when Dark, Pastel, Demi, and Mahogany were used as both sires and dams is shown in Figures c, and d. In the case of Dark coat color, 1.48% and 1.16% of Dark colored offspring were used when used as sires and dams of the same color respectively (Fig. 2c, and d). Similarly, Pastel color when used as sires produced 4.19% and when used as dams produced 5.3% Pastel colored offspring (Fig. 3c, and d). However, these numbers were higher when Demi and Mahogany were used as sires and dams alternatively. When used as sires Demi offspring produced 10.93% and when used as dams produced 32.5% of Demi offspring (Fig. 4c, and d). Approximately similar number of offspring were recorded when Mahogany colored sires (18.16%) and dams (18.19%) were used (Fig. 5c, and d).

Due to incomplete information about the color of either sire or dam and sometimes both, the color inheritance pattern of some individuals was not accurately identified. These individuals that were considered as “color not identified in sire or dam or both” group, accounted for 18.23% of Dark color, 12.09% of Pastel color, 13.82% of Demi color, and 10.39% of Mahogany color offspring.

When the same-colored parents were mated, the production of other colored offspring was found to be the highest for Demi and Mahogany. These findings suggest that among all four colors, at least two (Demi and Mahogany) were heterozygous. Similarly, the ancestral background also proposed that these colors had diverse colored ancestors until four generations. To verify this hypothesis, the breeding results of crossing (A × B) and reciprocal crossing (B × A) of Dark, Pastel, Demi, and Mahogany were performed, and the results are shown in Table 1. The statistically significant results (*P* value less than 0.05) shown in Table 1 demonstrated that the genes responsible for Dark, Pastel, Demi, and Mahogany colors have met the expected ratios (1:1 and 3:1) and thus can be considered heterozygous.

**Table 1:** Crossing of sire and dam with alternate colors and reciprocal crossing along with the production of offspring. The table also shows the *P*-value for the expected ratios of heterozygosity 1:1 and 3:1

Sire color	Dam color	Offspring color		*P*-value for 1:1	*P*-value for 3:1
		**D**	**NON-D**		
D	NON-D	134	650		
NON-D	D	106	470	< 0.00002	< 0.00001
		**P**	**NON-P**		
P	NON-P	215	747		
NON-P	P	272	609	< 0.00002	< 0.00001
		**DE**	**NON-DE**		
DE	NON-DE	470	340		
NON-DE	DE	1400	860	< 0.00002	< 0.00001
		**M**	**NON-M**		
M	NON-M	609	844		
NON-M	M	610	499	< 0.00002	< 0.00001

The ancestral background revealed that the ancestors of all four colors’ offspring were sometimes limited to a specific color and, most of the time, were diverse. Few individuals of Dark and Pastel were produced by mating sire and dam of the respective colors only (pure cross). On the other hand, Demi and Mahogany color mink ancestors were remarkably diverse in their background.

These results exhibiting the production of offspring from diverse colored ancestors demonstrated that the inheritance of coat color was considerably more than the involvement of a few sets of genes.

## Discussion

We found that mating sire and dam of the same color produced offspring of variety of colors. However, the probability of obtaining offspring of the same color as that of the parents was over 75% for all four-color types. This study also showed that using one of the parents with the desired color in offspring will probably result in the production of that color. These findings were further verified by retracing the pedigree up to four generations.

The mating of same-color parents (Dark and Pastel) produced Dark offspring at 99.38%, and Pastel offspring at 98.35% of the time. A study on Pastel coat color inheritance in sables *(Martes zibellina)* also showed that the mating of both parents of Pastel color produced offspring with Pastel fur coats only [17]. The breeding results of green-eyed Pastel individuals in Ontario, Wisconsin in 1941 showed that using the same color for sire and dam resulted in the production of only green-eyed Pastel phenotypes [19]. On the same farm, when green-eyed Pastel was crossed with Dark individuals, both Dark and green-eyed Pastel color individuals were observed [19]. Similarly, offspring produced by crossbreeding Pastel and black sables followed the 1:1 ratio corresponding to Mendelian inheritance [17]. The heterozygous nature of alleles responsible for Pastel color was also reported by Eklund et al. [26] and Shackelford [19] while crossing Ebony and Pastel color sire and dam respectively. It was concluded that all Pastel-colored mink in the ranch in Wisconsin originated from the heterozygous pairs [27]. Similarly, we also observed that crossing Pastel color individuals, irrespective of sex, with individuals of any other color followed both 1:1 and 3:1 ratios suggesting that Pastel color individuals produced by crossbreeding are indeed heterozygous.

Since the beginning of the domestication of wild mink, Dark color pelts have always been in high demand and are known as the standard color. These standard minks are almost Dark in color and is considered genetically a dominant color [4, 28]. This explained the highest number of Dark color offspring produced (99.38%) when Dark color was characteristic of both sires and dams in our study. To the best of our knowledge, there is no report about the crosses determining the genetic component underlying Dark color. Other than pure crossing (sire and dam of the same color), crossbreeding of Dark color with other color types was frequently practiced in mink breeding to produce new color phases such as Mahogany [12]. The heterozygous genes responsible for Dark color is demonstrated in our research where both 1:1 and 3:1 ratio was followed in the respective crosses analyzed.

In the case of Demi and Mahogany, the mating of sires and dams of the same color produced 14.76% and 22.41% of other color offspring rather than Demi and Mahogany respectively. Additionally, the production of Demi and Mahogany colored offspring in the pedigree when neither parent was of the same color revealed that both Demi and Mahogany colors might be produced as the result of crossbreeding and their heterozygous nature. The pedigree file analysis of the ancestors aided in the findings that the antecedents of the present Demi and Mahogany color in mink were from various colorations. The genomic studies in the same population using the whole genome sequence (WGS) data characterized Demi and Mahogany as highly admixed color types with observed heterozygosity of 31.12% and 30.93%, respectively [11].

The origin of Mahogany color in mink is attributed to the crossbreeding of brown and black mink lines which justifies its higher heterozygosity level [4]. The exact origin of Demi color mink is not mentioned in the literatures, but it is believed that crossbreeding might have resulted in coloration. Moreover, it has been evident that Demi and Mahogany color types have small genetic distances [11]. The small genetic distance can be attributed to the common ancestors in recent generations [29]. To further investigate this, the comparison of Demi and Mahogany colors within themselves was conducted. It was shown that the crossing of Demi and Mahogany irrespective, of sex, produced the offspring of either color notably. Furthermore, mating sire and dam of Demi color produced Mahogany color offspring 8.39% of the time. This scenario was also noted while Mahogany-colored sires and dams resulted in Demi-colored offspring 19.55% of the time (Fig. 4b, 5b). This might signify that the genes responsible for both of these colorations might be pleiotropic.

## Conclusion

The overall crosses analyzed provided valuable insight into the color type of parents which could be selected to produce offspring with desired coat colors. In general, mating parents with the same color results in the production of offspring with the same color most of the time. Similarly, using either sire or dam with the same desired color in offspring may also be advisable. However, the production of a specific color in offspring when none of the parents are of the same color as the offspring demonstrated that the inheritance of coat color is complicated. Furthermore, heterozygous genes responsible for color type have assisted the understanding of color inheritance in American mink. Even though coat color is termed a qualitative trait [30], the mutation of genes responsible for coat color and pleiotropic effect on morphological and physiological traits has made the genomic study of color inheritance in American mink necessary.

## Supplementary Information

**Additional file 1:** **Table S1.** Total number of mink (all eleven colors) in the pedigree along with number of mink with complete record of ancestral coat color until four generations. **Table S2. **Detail results showing the number of offspring produced when sires of Dark color were crossed with dams of 10 different color types and dams of Dark color were crossed with sires of 10 different color types. **Table S3.** Detail results showing the number of offspring produced when sires of Pastel color were crossed with dams of 10 different color types and dams of Pastel color were crossed with sires of 10 different color types. **Table S4.** Detail results showing the number of offspring produced when sires of Demi color were crossed with dams of 10 different color types and dams of Demi color were crossed with sires of 10 different color types. **Table S5.** Detail results showing the number of offspring produced when sires of Mahogany color were crossed with dams of 10 different color types and dams of Mahogany color were crossed with sires of 10 different color types. **Table S6.** Number and color type of offspring produced by crossing sire and dam of the same color. **Table S7.** Number of offspring produced by crossing and reciprocal crossing of all four-color types.

## Data Availability

The dataset supporting the conclusions of this article is included within the article and is named as Additional file 1.
